# Detection of Herbal Combinations and Pharmacological Mechanisms of Clinical Prescriptions for Coronary Heart Disease Using Data Mining and Network Pharmacology

**DOI:** 10.1155/2021/9234984

**Published:** 2021-10-23

**Authors:** Siling Bi, Liang Xu, Shouqiang Chen, Shuai Bu, Yunsheng Xu

**Affiliations:** ^1^Shandong University of Traditional Chinese Medicine, Jinan, China; ^2^The Second Hospital Affiliated to Shandong University of Traditional Chinese Medicine, Jinan, China

## Abstract

Though widely used in the treatment of coronary heart disease (CHD), the mechanism of traditional Chinese medicine (TCM) is still unclear because of its complex prescription rules. This study prospectively collected 715 prescriptions of TCM for the treatment of CHD. The characteristics of TCM in prescriptions were described and analyzed, and the rules of prescriptions were analyzed by using association rules. Frequency statistics showed that the high-frequency herbs with a frequency of more than 60% were Gan-cao, Huang-qi, Dang-gui, Chuan-xiong, Yan-hu-suo, and San-qi. The high-frequency herb combinations were summarized by using association rules. By using the method of the “Top *N* groups” to excavate the empirical prescriptions, the basic prescriptions for treating CHD were summarized. We named the intersection herbs of the basic prescriptions and the high frequency herbs as the core herbal prescription. To explore the possible mechanisms underlying the anti-CHD effect of the core herbal prescription, the bioactive components of core herbal prescription and their targets were screened out by using network pharmacology. Molecular docking was performed between the bioactive components and core targets. A total of 28 potential active ingredients and 5 core targets were identified for the treatment of CHD with core herbal prescription. The enrichment analysis results indicated that the mechanism of action mainly involved neuroactive ligand-receptor interaction and calcium signaling pathway. The commonly used herbal pairs for CHD with qi deficiency and blood stasis syndrome were Huang-qi and Dang-gui. The mechanism of action of common herbal pairs was also studied by network pharmacology. This study summarized the prescription rule of TCM in the treatment of CHD and may provide a new idea for the treatment of CHD.

## 1. Introduction

Coronary heart disease (CHD) is a common heart disease and one of the leading causes of death worldwide [1, 2]. The World Health Organization (WHO) estimates that there are approximately 17.9 million deaths from cardiovascular diseases (https://www.who.int/health-topics/cardiovascular-diseases) and 7.4 million deaths from CHD worldwide each year [3]. As the world's biggest killer, ischemic heart disease accounts for 16% of the world's total deaths. Since 2000, the largest increase in deaths has been for this disease, rising by more than 2 million to 8.9 million deaths in 2019 (https://www.who.int/news-room/fact-sheets/detail/the-top-10-causes-of-death).

TCM has a history of treating CHD and related diseases for thousands of years. It has unique advantages and remarkable clinical efficacy [4]. Due to its multicomponent characteristics, diseases can be treated through a variety of ways and targets [5, 6]. The large-scale clinical data are the core empirical knowledge source of TCM research [7]. Therefore, it is significant to study the experience of TCM in the treatment of CHD.

With modern computer technology shedding light on new ways for the innovation of TCM, quantitative research methods have attracted more and more attention. The adopted tools mainly consist of text mining, knowledge discovery, TCM database technology, data mining, and drug discovery through TCMSP [8]. The establishment of a database which contains the relationships between TCM Symptoms, TCM herbs, and modern medicine (MM) symptoms integrates TCM with modern medicine at both the phenotypic and molecular levels [9]. Data mining can analyze the prescription patterns of TCM from clinical data and find the potential relationship between herbs and diseases [10]. It can evaluate the effect of interventions in a real clinical setting, which opens up a new path for researches of integrative herb in CHD [11].

In recent years, with the rapid development of bioinformatics, network pharmacology has become a new and powerful means to systematically reveal the principles and functions of complex biological systems [12, 13], and it has become an effective method to initially observe the effects of TCM at the system level and explore the potential pharmacological mechanism of TCM at the molecular level [13, 14].

Network pharmacology can form a complex interaction network based on target molecules, biological functions, and active compounds, which is in line with the natural characteristics of TCM prescriptions and can systematically clarify the mechanism of action of TCM prescriptions at the molecular level. It is expected to be a promising overall strategy for the research of TCM prescriptions [15–17].

This study aims to summarize the prescriptions and medication rules of TCM in the treatment of CHD and analyze its action pathway. Based on the website of prominent TCM doctors, this study conducted data mining on the proven records of the treatment of CHD by prominent TCM doctors. Our study may provide a reference for the clinical use of TCM in the treatment of CHD and the analysis of its potential mechanism of action.

## 2. Materials and Methods

### 2.1. Prescription Source

The cases were searched by using the website of prominent TCM doctors (https://121.42.193.121/) and setting the subject words as “coronary heart disease” and “western herb diagnosis.” The time was set to be unlimited and the remaining conditions were set to default options. Finally, a total of 715 related cases were retrieved.

#### 2.1.1. Inclusion Criteria

① Clinical studies of TCM prescription in the treatment of CHD, including case analysis and control tests, were included. ② Cases with clinical medication experience of TCM and clear prescription composition were also included. ③ Patients with follow-up records should take the first prescription, and repeat prescriptions should be taken only once.

#### 2.1.2. Exclusion Criteria

① Cases with vague prescription symptoms and drug composition and/or no obvious therapeutic effect were excluded. ② Repeated cases were also excluded.

### 2.2. Prescription Screening and Standardization of TCM Terminology

Through the above setting of a series of conditions, a total of 715 prescriptions including 182 Chinese herbs were included. In order to ensure the rationality of data analysis and the accuracy of Chinese herbs, the names of herbs were standardized with reference to Chinese Pharmacopoeia 2015 Edition.

### 2.3. Data Statistics and Analysis

#### 2.3.1. Frequency Statistics

The use frequency of herbs involved in the prescriptions included in the system was ranked in descending order, and the use frequency of the four qi, five flavors, and meridians of herbs were also ranked in descending order. The results were derived separately.

#### 2.3.2. Analysis of Association Rules of Commonly Used Herbs in Basic Prescriptions

To generate association rules more effectively, the Apriori algorithm was used in data mining. In the analysis of medical records, it was possible to mine the association rules between Chinese herbs in the medical records of prominent TCM doctors and mine their high-frequency herb combinations. Then, the “Top *N* group” method was used to excavate experiential prescriptions and summarize the basic prescriptions for the treatment of CHD by prominent TCM doctors.

#### 2.3.3. Study on Medication of Qi Deficiency and Blood Stasis Syndrome

According to TCM, qi deficiency and blood stasis are the fundamental and pathological basis of CHD. Clinical case review, cross-sectional investigation, and systematic literature review showed that qi deficiency and blood stasis syndrome are the most common clinical syndrome types of CHD and were the core syndromes throughout the occurrence and development of CHD [18]. The common herbal pairs for qi deficiency and blood stasis syndrome were found by association rules.

### 2.4. Analysis of Pharmacological Mechanism

With the aid of the Chinese medicine system pharmacology analysis platform (TCMSP, https://tcmspw.com/tcmsp.php), the chemical components of core herbal prescription were obtained. By using the compounds with bioavailability (OB) ≥30% and drug-like properties (DL) ≥ 0.18 as candidate active ingredients, the targets corresponding to the active ingredients were obtained by using the UniProt database (https://www.Unitprot.org/).

With “coronary heart disease” as the keyword, the protein targets of CHD were obtained through the GeneCards database. Then, using these targets as disease candidate targets, the gene names of the protein targets were queried in the UniProt database.

Wayne analysis was performed by Venny 2.1 software. The potential overlapping protein targets were obtained by intersecting the corresponding target of the drug candidate active ingredient with the single disease protein target. The protein network structure of potential intersection protein targets was visualized by Cytoscape 3.7.2 software.

The common targets of core herbal prescription and CHD were imported into the String protein interaction database (https://string-db.org/), and target protein–protein interaction (PPI) analysis was conducted. The whole node relationship information was exported, a target–target PPI network was constructed by using Cytoscape 3.7.2, and the core targets were screened out according to Degree.

In this study, DAVID software (https://david.ncifcrf.gov/) was used to perform GO enrichment analysis on the core target protein obtained after network merging. Statistical hypergeometric distribution was used to quantitatively evaluate protein populations. *P* value was used to reflect the significance of biological function of protein. Biological process, molecular function, and cellular component were selected. DAVID software was also used to carry out KEGG gene and signal pathway enrichment analysis on core protein target genes of drug component-disease intersection. Through the enrichment analysis of functional items, the main action pathways of core herbal prescription in the prevention and treatment of CHD were obtained.

### 2.5. Potential Active Ingredient-Target Molecular Docking Verification

Firstly, taking the mean parameters of “Degree” nodes in the component-target disease network model as reference conditions, the core active ingredients greater than the mean value were screened out. The MOL2 file of the relevant core active ingredients and 3D structure file of the key target protein (PDB format) were downloaded from TCMSP database and RSB database (https://www.rcsb.org), separately. Then, target protein files were imported into Chimera 1.14 software. Each protein molecule was minimally optimized and hydrodehydrated, and its primary ligand was removed. At the same time, the core active ingredients were introduced, and the treatment method was the same as above except for removing the mixture. Finally, after the performing of molecular docking by AutoDock-Vina 1.1.2 software, the obtained results were visualized.

## 3. Results

### 3.1. The Usage Frequency of Herbs

Among the 182 herbs, the top six high-frequency herbs were Gan-cao, Huang-qi, Dang-gui, Chuan-xiong, Yan-hu-suo, and Dang-gui. There were 27 herbs and 134 herbs that have been used more than 100 times and less than 50 times, respectively. The top 48 high-frequency herbs are shown in Table 1.

### 3.2. The Property, Taste, and Meridian Distribution of Herbs

**Figure 1:**
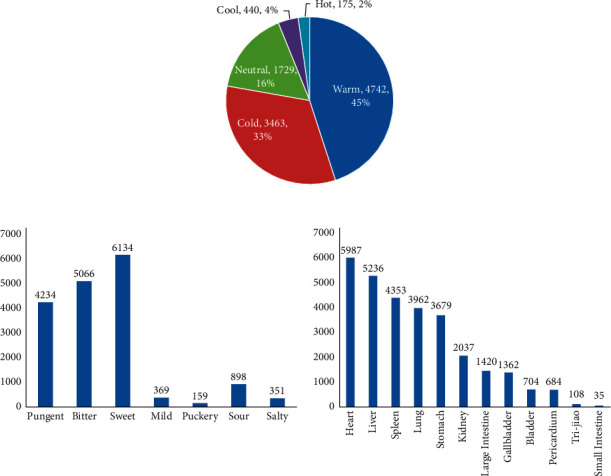
Frequency distribution of the property, taste, and meridian distribution.

The statistical results showed that among the four properties attributes, warm herbs were the most frequently used, accounting for 44.95%. Also, among the five flavor attributes, sweet was the most frequently used, accounting for 58.15%. The heart and liver meridians accounted for 56.75% and 49.64%, respectively (Table 2 and Figure 1).

### 3.3. Association Rules Analysis of High-Frequent Herbal Combination

The Apriori algorithm was used for the first scan, and the candidate set named C_1_ was obtained, that is, all herbs and their frequencies in the case of TCM for the treatment of CHD. Then, the minimum support was set to 0.3 to delete the candidate set with a frequency lower than 0.3, and finally the frequent set named *L*_1_ was obtained [19] (Table 3).

The candidate set named *C*_2_ was obtained from each combination of Chinese medicines in *L*_1_. Combinations with support less than 0.3 in candidate set named *C*_2_ were deleted to get *L*_2_, that is, the combination of two herbs (Table 4).

*L*_3_, *L*_4_, and *L*_5_ were obtained by analogy and are shown in Tables 5–7, respectively.

### 3.4. The Core Groups and Basic Prescriptions

There were seven combinations of five Chinese herbs, involving seven Chinese herbs (Table 7). It was not difficult to find that the last six combinations all evolved from the first combination. From this, you can refer to the “Top *N* groups” method of mining empirical prescriptions [19]. That is, the corresponding minimum support was set to display the five-flavored herb combination, and they were arranged in the order of more-flavored herb combination to the less-flavored herb combination. Among the combinations of five flavors of herb, the combination with the highest frequency was the basic prescription for doctors to add or subtract. They were the motherboard for us to excavate the basic prescriptions. We selected the first “*N*” group in order, found out the Chinese herb that was different from the mother board as the daughter board and added it to the mother board. In this way, the basic prescriptions could be summarized. From this, we discovered that the basic prescriptions of TCM for the treatment of CHD were “Gan-cao, Huang-qi, Mai-dong, San-qi, Yan-hu-suo, Wu-wei-zi, and Dang-gui.”

### 3.5. Discovery of Commonly Used Herbal Pairs

The commonly used herbal pairs were obtained by setting the subject words as “coronary heart disease” and “western herb diagnosis” and the syndromes as “qi deficiency” and “blood stasis” and the minimum support to 0.6. Finally, a total of 372 related cases were retrieved. The commonly used herbal pairs for CHD with qi deficiency and blood stasis syndrome were Huang-qi and Dang-gui (Table 8).

### 3.6. Pharmacological Mechanisms of Core Herbal Prescription

**Figure 2:**
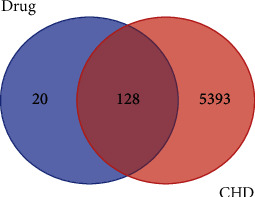
Common targets for drugs and diseases.

The top six high-frequency herbs were Gan-cao, Huang-qi, Dang-gui, Chuan-xiong, Yan-hu-suo, and Dang-gui (Table 1). From the “Top *N* groups” method (Section 3.4), we discovered that the basic prescriptions of TCM for the treatment of CHD were “Gan-cao, Huang-qi, Mai-dong, San-qi, Yan-hu-suo, Wu-wei-zi, and Dang-gui.” We named the intersection herbs of the high-frequency herbs and the basic prescriptions as the core herbal prescription containing Gan-cao, Huang-qi, Yan-hu-suo, San-qi, and Dang-gui. To investigate the pharmacological mechanisms of the herbs involved in the core herbal prescription, we further obtained the ingredients of these five herbs from TCMSP with the filtering of pharmacokinetic parameters of OB ≥ 30% and DL ≥ 0.18. In total, we finally obtained 158 ingredients for these five herbs. In particular, 93 active ingredients of Gan-cao, 20 active ingredients of Huang-qi, 8 active ingredients of San-qi, 49 active ingredients of Yan-hu-suo, and 2 active ingredients of Dang-gui were obtained. Among them, quercetin was shared by Gan-cao, Huang-qi, Yan-hu-suo, and San-qi. Mairin, Jaranol, isorhamnetin, formononetin, Calycosin, and kaempferol were shared by Gan-cao and Huang-qi. Sitosterol was shared by Gan-cao and Yan-hu-suo. Beta-sitosterol was shared by Dang-gui and San-qi. Stigmasterol was shared by Dang-gui, Yan-hu-suo, and San-qi. Next, we obtained 148 drug targets for these five herbs referring to the TCMSP database, which has 128 shared targets with the gene list of CHD (Figure 2).

**Figure 3:**
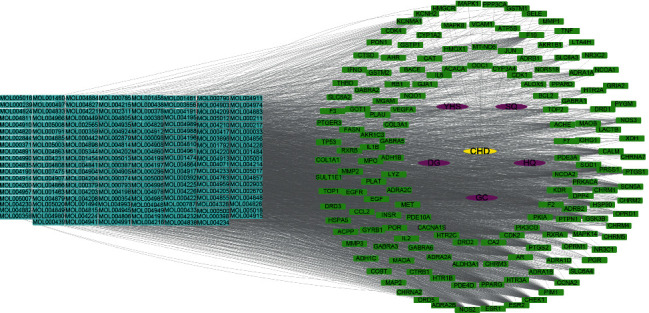
Herb ingredient-target-disease network. Yellow represents the disease, purple represents the herb, blue represents the active ingredient of the herb, and green represents the related targets of CHD.

The active ingredient and corresponding target information were imported into Cytoscape 3.7.2 software to draw the interaction network diagram, and the herb ingredient-target-disease core action network was extracted. The network topology analysis results showed that there were 321 network nodes, the network concentration degree was 0.372, the network density was 0.080, the network heterogeneity was 1.175, and the shortest path was 102720 (100%). The shape, size, and color of the diseases, herbs, targets, and ingredients were adjusted, and the target interaction relationship was visualized (Figure 3).

**Figure 4:**
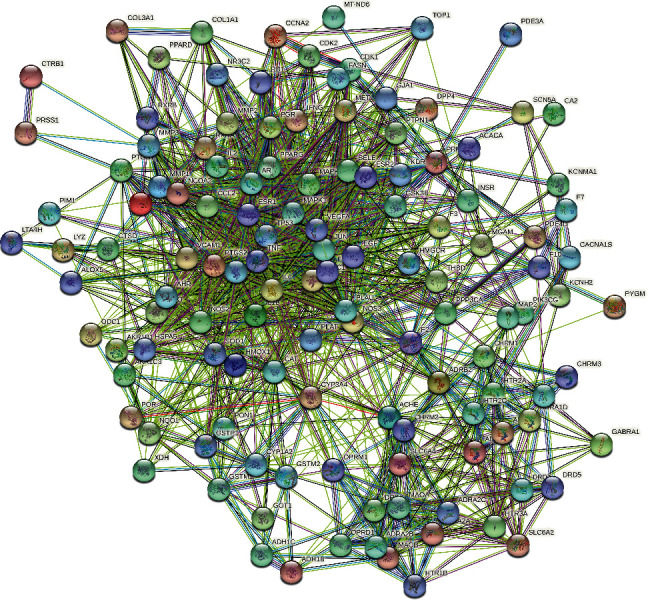
Interaction diagram between herbs and potential targets of CHD.

**Figure 5:**
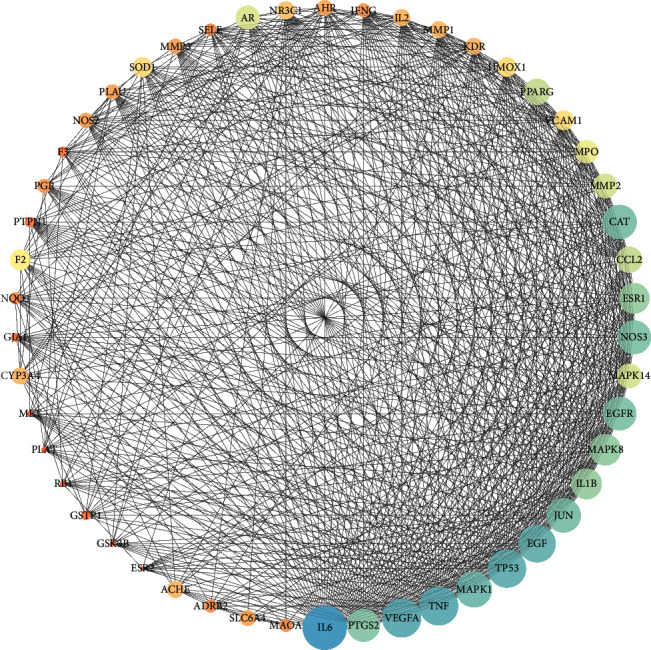
PPI network diagram.

The common target of core herbal prescription and CHD were imported into the String database. With *Homo sapiens* as the limited condition, protein interactions were obtained by using the multiple protein tool. The result involved a total of 128 nodes, 1321 edges, and an average node degree of 20.6, and the average local clustering coefficient was 0.552. As shown in Figure 4, the full node relationship information was exported and then imported into Cytoscape, and the core genes of PPI network were screened out according to the Degree value for visualization processing. As shown in Figure 5, the top five nodes with degree values were IL6, PTGS2, VEGFA, TNF, and MAPK1. These five key targets may play an important role in the treatment of CHD with core herbal prescription.

**Figure 6:**
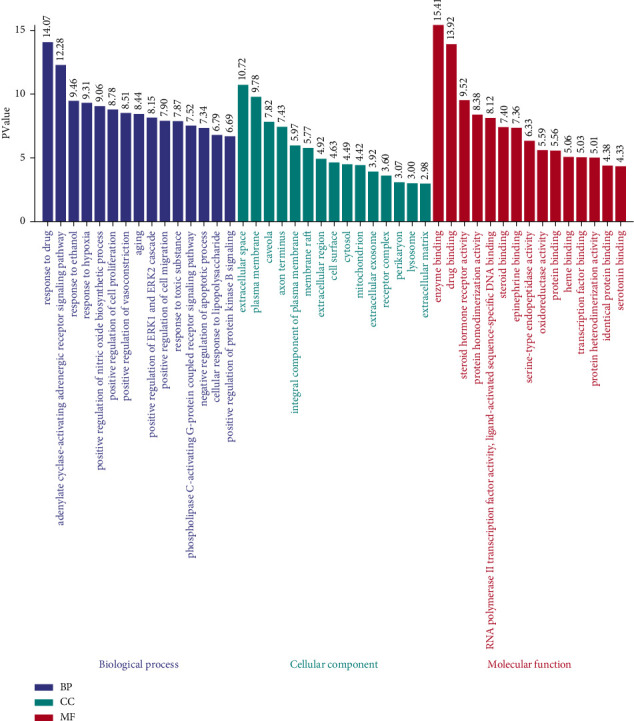
GO pathway analysis (the top 15).

Enrichment analysis of 128 common targets of core herbal prescription and CHD was performed by DAVID software. 132 GO biological processes, 19 GO cell components, and 36 GO molecular functions were obtained. The biological processes included response to drug, adrenergic receptor signaling pathway, response to ethanol, response to hypoxia, and positive regulation of nitric oxide biosynthetic process. The cell components included extracellular space, plasma membrane, caveola, axon terminus, and integral component of plasma membrane. The molecular functions included enzyme binding, drug binding, steroid hormone receptor activity, protein homodimerization activity, RNA polymerase II transcription factor activity, and ligand-activated sequence-specific DNA binding. The top 15 of them were selected by Excel to carry out the three-in-one histogram visualization processing (Figure 6).

**Figure 7:**
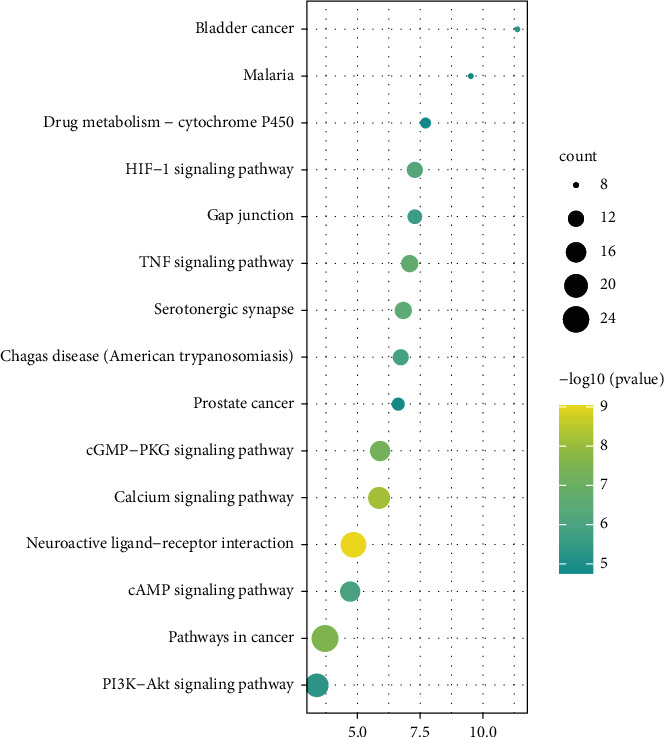
KEGG pathway analysis (the top 15).

104 pathways were obtained by KEGG pathway analysis, including neuroactive ligand-receptor interaction, calcium signaling pathway, pathways in cancer, cGMP-PKG signaling pathway, and TNF signaling pathway. The top 15 results of KEGG signal enrichment analysis were visualized by using the WeiShengXin (https://www.bioinformatics.com.cn/) online mapping website (Figure 7).

### 3.7. Results of Molecular Docking between Potential Active Ingredients and Key Target Proteins

**Figure 8:**
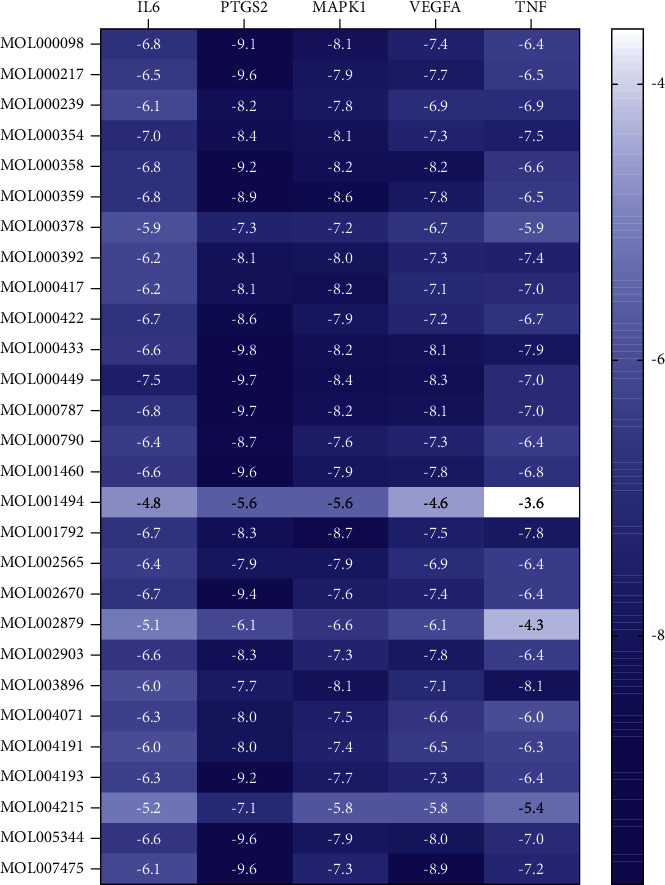
Binding energy of herbs and disease targets.

**Figure 9:**
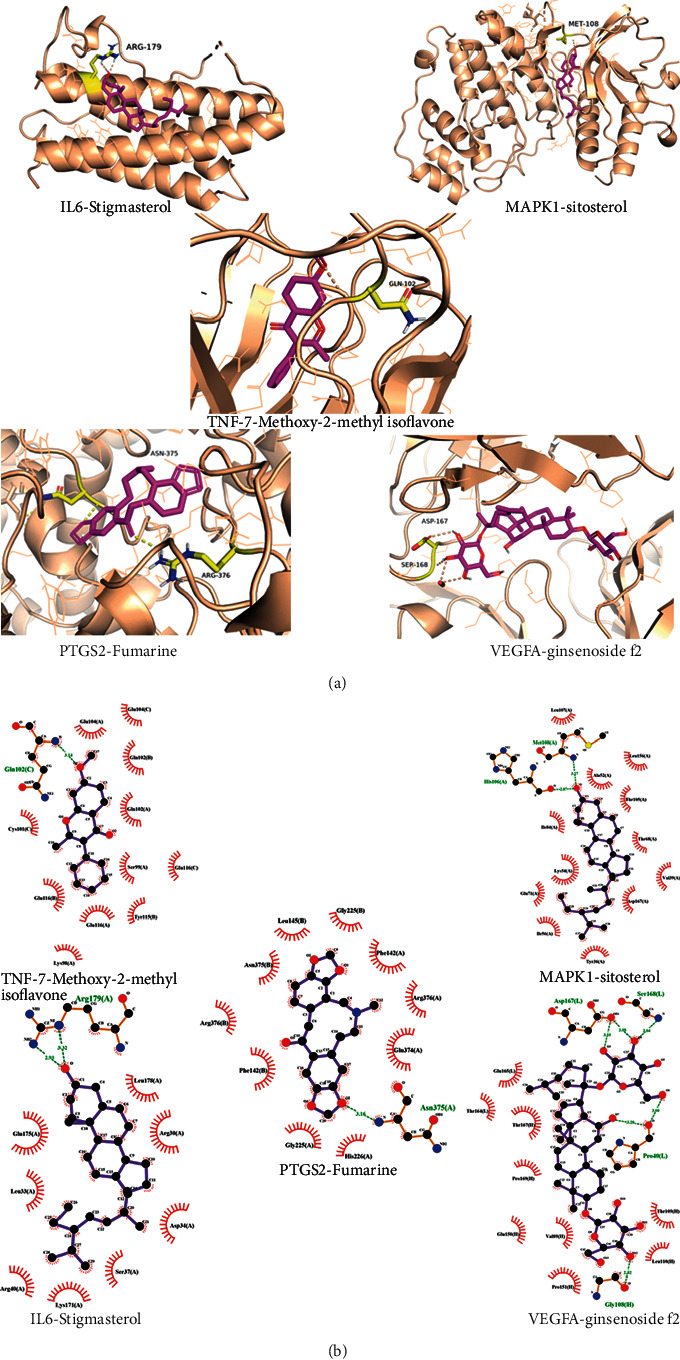
Molecular docking stereogram (a) and molecular docking molecular diagram (b).

Through the analysis of the “Degree” parameter in the ingredient-target-disease network model, 28 active compounds were screened out. Studies have shown that the lower the binding energy required for ligands for the binding of the ligand to receptors, the more stable the binding conformation. The results of this study showed that the binding energy between the target of core herbal prescription and CHD was negative. It is generally believed that the binding energy less than −4.25 kcal mol^−1^ indicates that the ligand and the receptor have a certain binding activity. Binding energy less than −5.0 kcal mol^−1^ indicates good binding activity while binding energy less than −7.0 kcal mol^−1^ indicates strong binding activity [20]. The results of molecular docking are shown in Figure 8, and the docking mode of the compound with strong binding activity and the target protein molecule is shown in Figure 9.

### 3.8. Network Pharmacology Research on Herbal Pairs

According to the pharmacokinetic parameters of OB ≥30% and DL ≥0.18, 20 active ingredients of Huang-qi, 2 active ingredients of Dang-gui were obtained. There were 22 active ingredients in total (Table 9). Target prediction was carried out through the TCMSP database, and 133 effective targets of herbal pairs were obtained after correction with UniProt ID number.

**Figure 10:**
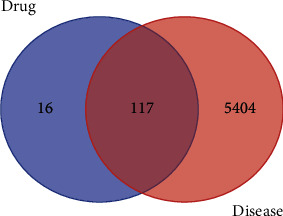
Common targets for drug pairs and diseases.

Wayne analysis was carried out by Venny 2.1 software, and 117 intersection targets of herbs with CHD were screened out (Figure 10).

**Figure 11:**
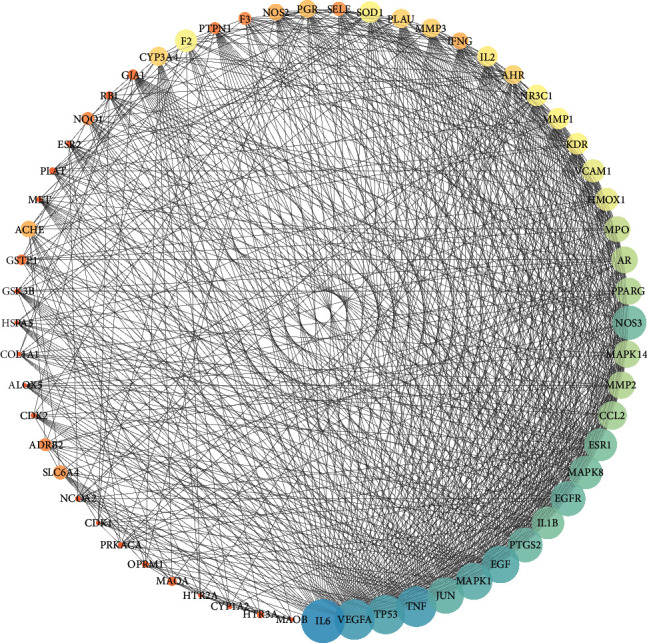
PPI network diagram.

The full node relationship information was exported and then imported into Cytoscape, and the core genes of PPI network were screened out according to the Degree value for visualization processing. As shown in Figure 11, the top five nodes with degree values were IL6, VEGFA, TP53, TNF, and JUN.

**Figure 12:**
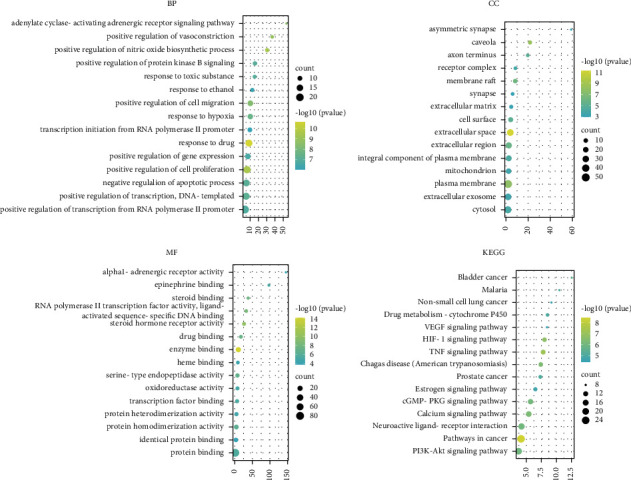
GO and KEGG pathway analysis (the top 15).

The DAVID database was used to perform gene GO function and KEGG enrichment analysis on the intersection targets. GO analysis results showed that the main biological processes involved in the treatment of CHD with qi deficiency and blood stasis syndrome by Huang-qi and Dang-gui include response to drug, positive regulation of cell proliferation, extracellular space, caveola, enzyme binding, and steroid hormone receptor activity. The pathways involved include TNF signaling pathway, pathways in cancer, HIF-1 signaling pathway, Chagas disease (American trypanosomiasis), and calcium signaling pathway (Figure 12).

## 4. Discussion

CHD is thought to be a result of “congenital deficiency and postnatal malnutrition.” Deficiency in origin includes deficiency in qi, blood, yin, and yang; excess in superficiality includes qi stagnation, blood stasis, phlegm, and cold coagulation [21]. The previous epidemiological studies found that the core syndrome of patients with CHD is “Qi Deficiency and Phlegm-Blood Stasis” [22]. Contemporary famous old TCM doctors focus on certain core drugs when treating patients with CHD. Based on the top 3 most commonly used drug categories, the main therapeutic approaches used by these prestigious doctors included “Tonifying Qi, Activating Blood, and Eliminating Phlegm” [23].

This study collected 715 prescriptions including 182 Chinese herbs. The characteristics of TCM in prescriptions were described and analyzed, and the rules of prescriptions were analyzed by using association rules. Frequency statistics showed that the high frequency herbs with a frequency of more than 60% were Gan-cao, Huang-qi, Dang-gui, Chuan-xiong, Yan-hu-suo, and San-qi. At the same time, we also innovatively put forward the “Top *N* groups” empirical prescription mining method to summarize the basic prescriptions of TCM for the treatment of CHD with the aid of Apriori algorithm. We discovered that the basic prescriptions of TCM for the treatment of CHD were “Gan-cao, Huang-qi, Mai-dong, San-qi, Yan-hu-suo, Wu-wei-zi, and Dang-gui.” We named the intersection herbs of the basic prescriptions and the high-frequency herbs as the core herbal prescription. We found that Gan-cao, Huang-qi, Yan-hu-suo, San-qi, and Dang-gui were core herbal prescription. They can alleviate CHD from multiple angles. Gan-cao has the effects of tonifying the middle body and supplementing Qi [24]. Gan-cao has protective effects on the heart, and the hydrolysates of its main component glycyrrhizin is glycyrrhetinic acid, which has the effect of intervening the permeability transition of rat heart mitochondria [25]. The extract of Gan-cao can antagonize the cardiotoxicity induced by DOX and maintain the normal function of cardiac myocytes [26]. Huang-qi plays a role in improving metabolism, digestion, and endocrine function of the body [10]. Dang-gui nourishes blood and promotes blood circulation, Yan-hu-suo, and San-qi can promote blood circulation and relieve pain. Notoginsenoside R1 exerts cardioprotective effects against diabetic cardiomyopathy through its inhibition of oxidative stress and apoptosis [27, 28]. Pharmacodynamics studies showed that Panax notoginseng saponins (PNS) generated an obvious antiatherosclerosis action [29]. The application of core herbal prescription has confirmed that “Tonifying Qi, Nourishing Yin, Activating Blood, and Eliminating Phlegm” are the most commonly used therapeutic methods for patients with CHD [23]. Taking “Qi Deficiency” and “Blood Stasis” as the retrieval conditions, the commonly used drug pairs for CHD with qi deficiency and blood stasis syndrome were Huang-qi and Dang-gui. From this point of view, we can treat common CHD based on syndrome differentiation by using the core herbal prescription of TCM combined with the rules of medication. The application of data mining technology enables the experience of TCM to be passed on.

Based on network pharmacology, this study screened the effective active ingredients and targets of core herbal prescription of TCM in the treatment of CHD. The intervention effect and mechanism of core herbal prescription on CHD were systematically studied. Through PPI network analysis, we found that the degree of value in the top five targets were IL6, PTGS2, VEGFA, TNF, and MAPK1. These five key targets may play an important role in the treatment of CHD with core herbal prescription.

IL-6 is independently associated with the risk of major adverse cardiovascular events, cardiovascular and all-cause mortality, myocardial infarction, heart failure, and cancer mortality in patients with stable CHD [30].

In the pathological process of CVD, cardiovascular disease stimulates the expression of inflammatory cytokines, leading to the upregulation of inflammatory mediators such as PTGS-2 [31, 32], which promotes PTGS-2 gene to encode COX-2 protein, and finally resulting in vascular calcification [33, 34].

VEGF is a homodimeric protein, including VEGFA, VEGFB, VEGFC, VEGFD, and placental growth factor (PLGF). VEGFA can induce acute, persistent and chronic leakage [35, 36], involving remodeling of junctions, induction of fenestrations, vesicle-vacuum organelles (VVOs), or transcellular transcytosis. Evidences showed that abnormal changes in the expression of certain genes in coronary artery disease play a crucial role in the pathogenesis of atherosclerosis [37, 38]. MicroRNAs are short noncoding RNAs with regulatory functions that are widely found in animals and plants [39]. Previous studies have found that miR-451 can inhibit liver tumor angiogenesis, suggesting that it may be involved in the formation of blood vessels [40]. Comprehensive bioinformatics prediction and dual-luciferase reporter gene detection showed that VEGFA is the target gene of miR-451b. MiR-451b may affect the PI3KAkt-mTOR signaling pathway by altering the expression of VEGFA, thereby regulating the proliferation and apoptosis of HUVEC, and ultimately participating in the occurrence and development of CHD [41].

As a member of the TNF receptor family, the transmembrane death receptor induces apoptosis through an exogenous pathway [42]. TNF-*α*/TNFR1 complex is involved in exogenous apoptosis of cardiomyocytes [43, 44]. Several typical apoptosis-stimulating fragment ligands (FasL)/FasR, TNF-*α*/TNFR1, TNF-associated apoptosis-inducing ligand (TRAIL)/DR4 and TRAIL/DR5, and their corresponding death receptors can transmit death signals from cell surface to intracellular pathways through the death domain [42].

Atherosclerosis is a systemic degenerative inflammatory vascular disease and the primary underlying cause of CHD [45]. Based on the results of bioinformatics and cloning studies, researchers found that about 50 circulating miRNAs were associated with cardiovascular disease [46]. Many studies have proved that miRNAs (miR-1, miR133a, and miR-133b) play an important role in heart injury and myocardial infarction [47]. Mitogen-activated protein kinase 1 (MAPK1), as one of the mRNAs, is a candidate target gene for a variety of miRNAs [48]. Studies have shown that miR-197 leads to the silencing of MAPK1 gene by recognizing and specifically binding to the predicted site of the untranslated region of MAPK1 mRNA 3 [49]. Multiple studies have shown that MAPK1 plays an important role in the occurrence and development of atherosclerotic lesions [50–52]. A previous study has shown that everolimus-induced mTOR inhibition can selectively depletes macrophages in atherosclerotic plaques by autophagy, and mTOR pathway can stimulate macrophage autophagy and prevent atherosclerotic plaque formation [53–55]. Zhu et al. examined the interaction between miR-15b-5p and MAPK1, and the results showed that MAPK1 was a functional target for miR-15b-5p to regulate the progression of CAD. Long noncoding RNA (lncRNA) MALAT1 triggers the mTOR signaling pathway by regulating miR-15b-5p and MAPK1 [56].

132 GO biological processes, 19 GO cell components and 36 GO molecular functions were obtained by enrichment analysis. Biological processes included response to drug, adenylate cyclase-activating receptor signaling pathway, response to ethanol, response to hypoxia, and positive regulation of nitric oxide biosynthetic process. Cell components included extracellular space, plasma membrane, caveola, axon terminus, and integral component of plasma membrane. Molecular functions included enzyme binding, drug binding, steroid hormone receptor activity, protein homodimerization activity, RNA polymerase II transcription factor activity, and ligand-activated sequence-specific DNA binding.

A total of 104 pathways were obtained by KEGG pathway analysis, including neuroactive ligand-receptor interaction, calcium signaling pathway, pathways in cancer, cGMP-PKG signaling pathway, and TNF signaling pathway. Among these pathways, neuroactive receptor-ligand interaction has an influence on many organs and systems, including the nervous system, and may cause long-term dysfunction [57]. The calcium signaling pathway may influence the functions of nerves, muscles, and the heart through the release of neurotransmitters, muscular contraction, and cardiac conduction, respectively [58].

The results of molecular docking showed that the potential active ingredients of core herbal prescription have good binding properties with the intersection target protein of CHD. Based on this, we speculated that these components may play an important role in regulating the expression of CHD target proteins or interacting with these proteins.

Based on data mining, this research summarized the basic prescriptions and core herbal prescription of TCM in treating CHD. The network pharmacological research results found that core herbal prescription can achieve the anti-CHD effects through multicomponent, multitarget, and multisignal pathways. The results of molecular docking showed that the potential active ingredients of core herbal prescription had good binding properties with the intersection target protein of CHD, which confirmed that TCM treated CHD through multiple targets and pathways.

Although we have systematically studied the TCM treatment of CHD, there are several limitations of the study. Firstly, due to the limitation of data volume, the results of this study may be biased to some extent. Secondly, there are many methods of data mining for clinical prescriptions, involving association rule analysis, cluster analysis, artificial neural network, Bayesian algorithm, complex network, database method, and so on [59, 60]. However, our data mining method is relatively simple. Third, we only conducted network pharmacology research on the core herbal prescription, and the network pharmacology research on the successful prescriptions has not been done, for example, the relevant study of SheXiang XinTongNing (XTN) [61]. Related studies on the successful prescriptions need to be carried out in the future. Fourth, the relationships between diseases and syndromes are complex; one disease may express different syndromes [62]. Our research does not involve the distribution of syndromes. In future research, we will conduct research on the rules and mechanisms of the medication of syndromes. We will verify the effective targets and pathways through experiments to provide a strong basis for the development of new drugs.

## 5. Conclusions

A new prescription of CHD composed of Gan-cao, Huang-qi, San-qi, Yan-hu-suo, and Dang-gui has been extracted. The commonly used drug pairs for CHD with qi deficiency and blood stasis syndrome were Huang-qi and Dang-gui. The core herbal prescription mainly intervenes in neuroactive ligand-receptor interaction, calcium signaling pathway, pathways in cancer, cGMP-PKG signaling pathway, and TNF signaling pathway. Our results showed that the method and software used in this study can effectively analyze the mechanism and law of TCM prescription.

## Figures and Tables

**Table 1:** Frequency distribution of high frequency herbs.

No.	Herbs	Frequency	Percentage	No.	Herbs	Frequency	Percentage
1	Gan-cao	543	75.94	25	Gua-lou	102	14.27
2	Huang-qi	536	74.97	26	Qing-hao	101	14.13
3	Dang-gui	525	73.43	27	Qiang-huo	101	14.13
4	Chuan-xiong	509	71.19	28	Sha-ren	94	13.15
5	Yan-hu-suo	483	67.55	29	Dan-pi	93	13.01
6	San-qi	444	62.1	30	Chai-hu	92	12.87
7	Mai-dong	423	59.16	31	Huang-bo	90	12.59
8	Dan-shen	417	58.32	32	Rou-gui	89	12.45
9	Ye-ge-gen	372	52.03	33	Zi-shi-ying	89	12.45
10	Wu-wei-zi	361	50.49	34	Hou-pu	79	11.05
11	Huang-lian	333	46.57	35	He-shou-wu	70	9.79
12	Bing-pian	322	45.03	36	Shu-di	70	9.79
13	Suan-zao-ren	286	40	37	Ye-jiao-teng	67	9.37
14	Huang-qin	181	25.31	38	Zhi-zi	67	9.37
15	Gui-zhi	176	24.62	39	Xing-ren	65	9.09
16	Gou-teng	175	24.48	40	Zhi-qiao	63	8.81
17	Fu-ling	170	23.78	41	Ban-xia	62	8.67
18	Ze-xie	167	23.36	42	Jue-ming-zi	61	8.53
19	Shui-zhi	154	21.54	43	Da-huang	59	8.25
20	Mu-xiang	148	20.7	44	Xie-bai	58	8.11
21	Bai-shao	140	19.58	45	Fu-zi	56	7.83
22	Ren-shen	119	16.64	46	Bai-zhu	56	7.83
23	Yin-yang-huo	110	15.38	47	Dang-shen	53	7.41
24	Sheng-di	105	14.69	48	Xi-xin	51	7.13

**Table 2:** Frequency distribution of the property, taste, and meridian distribution.

Project	Property, taste, and meridian distribution	Frequency	Percentage
Four properties	Warm	4742	44.95
	Cold	3463	32.83
	Mild	1729	16.39
	Cool	440	4.17
	Hot	175	1.66

Five tastes	Pungent	4234	40.14
	Bitter	5066	48.02
	Sweet	6134	58.15
	Mild	369	3.5
	Puckery	159	1.51
	Sour	898	8.51
	Salty	351	3.33

Meridian distribution	Heart	5987	56.75
	Liver	5236	49.64
	Spleen	4353	41.26
	Lung	3962	37.56
	Stomach	3679	34.88
	Kidney	2037	19.31
	Large Intestine	1420	13.46
	Gallbladder	1362	12.91
	Bladder	704	6.67
	Pericardium	684	6.48
	Tri-jiao	108	1.02
	Small Intestine	35	0.33

**Table 3:** Frequent set *L*_1_.

Herbs	Support	Herbs	Support
Gan-cao	0.76	Dan-shen	0.58
Huang-qi	0.75	Ye-ge-gen	0.52
Dang-gui	0.73	Wu-wei-zi	0.51
Chuan-xiong	0.71	Huang-lian	0.46
Yan-hu-suo	0.68	Bing-pian	0.45
San-qi	0.62	Suan-zao-ren	0.40
Mai-dong	0.59		

**Table 4:** Frequent set *L*_2_.

Combination of TCM (2)	Confidence	Combination of TCM (2)	Confidence
Gan-cao, Huang-qi	0.62	Chuan-xiong, Mai-dong	0.4
Huang-qi, Dang-gui	0.59	Yan-hu-suo, Dan-shen	0.39
Gan-cao, Dang-gui	0.58	Yan-hu-suo, Bing-pian	0.39
Yan-hu-suo, San-qi	0.57	Yan-hu-suo, Wu-wei-zi	0.39
Dang-gui, Chuan-xiong	0.56	Dang-gui, Wu-wei-zi	0.39
Huang-qi, Mai-dong	0.56	San-qi, Dan-shen	0.38
Gan-cao, Chuan-xiong	0.54	San-qi, Wu-wei-zi	0.38
Gan-cao, Yan-hu-suo	0.53	Dang-gui, Ye-ge-gen	0.38
Huang-qi, San-qi	0.53	Huang-qi, Ye-ge-gen	0.37
Huang-qi, Yan-hu-suo	0.53	Yan-hu-suo, Ye-ge-gen	0.37
Huang-qi, Chuan-xiong	0.52	Mai-dong, Dan-shen	0.37
Gan-cao, Mai-dong	0.51	San-qi, Bing-pian	0.36
Huang-qi, Wu-wei-zi	0.5	Gan-cao, Ye-ge-gen	0.36
Gan-cao, San-qi	0.5	Chuan-xiong, Bing-pian	0.35
Dang-gui, Yan-hu-suo	0.49	Chuan-xiong, Wu-wei-zi	0.34
Chuan-xiong, Yan-hu-suo	0.49	Gan-cao, Huang-lian	0.33
Mai-dong, Wu-wei-zi	0.48	San-qi, Ye-ge-gen	0.33
Dang-gui, San-qi	0.46	Huang-qi, Suan-zao-ren	0.33
Dang-gui, Mai-dong	0.46	Dang-gui, Huang-lian	0.33
Huang-qi, Dan-shen	0.45	Gan-cao, Suan-zao-ren	0.33
Gan-cao, Dan-shen	0.45	Huang-qi, Bing-pian	0.33
Chuan-xiong, Ye-ge-gen	0.45	Yan-hu-suo, Huang-lian	0.32
Chuan-xiong, San-qi	0.44	Dan-shen, Wu-wei-zi	0.32
Dang-gui, Dan-shen	0.44	Gan-cao, Bing-pian	0.32
Gan-cao, Wu-wei-zi	0.44	Chuan-xiong, Huang-lian	0.31
Yan-hu-suo, Mai-dong	0.44	Dang-gui, Suan-zao-ren	0.31
San-qi, Mai-dong	0.42	Huang-qi, Huang-lian	0.31
Chuan-xiong, Dan-shen	0.4	Dang-gui, Bing-pian	0.3

**Table 5:** Frequent set *L*_3_.

Combination of TCM (3)	Confidence	Combination of TCM (3)	Confidence
Dang-gui, Gan-cao, Huang-qi	0.49	Dang-gui, Dan-shen, Gan-cao	0.36
Gan-cao, Huang-qi, Mai-dong	0.49	Chuan-xiong, Dang-gui, San-qi	0.35
Huang-qi, San-qi, Yan-hu-suo	0.48	San-qi, Wu-wei-zi, Yan-hu-suo	0.35
Huang-qi, Mai-dong, Wu-wei-zi	0.48	Chuan-xiong, Gan-cao, San-qi	0.35
Gan-cao, San-qi, Yan-hu-suo	0.46	Bing-pian, San-qi, Yan-hu-suo	0.35
Gan-cao, Huang-qi, San-qi	0.45	Dan-shen, Huang-qi, Mai-dong	0.34
Gan-cao, Huang-qi, Yan-hu-suo	0.45	Chuan-xiong, Dang-gui, Ye-ge-gen	0.34
Dang-gui, Huang-qi, Mai-dong	0.44	Chuan-xiong, Gan-cao, Mai-dong	0.34
Chuan-xiong, Dang-gui, Gan-cao	0.43	Dang-gui, Mai-dong, Yan-hu-suo	0.34
Chuan-xiong, Gan-cao, Huang-qi	0.43	Dan-shen, San-qi, Yan-hu-suo	0.33
Chuan-xiong, Dang-gui, Huang-qi	0.43	Chuan-xiong, Mai-dong, Wu-wei-zi	0.33
Gan-cao, Huang-qi, Wu-wei-zi	0.43	Chuan-xiong, Dang-gui, Mai-dong	0.33
Dang-gui, Huang-qi, Yan-hu-suo	0.42	Gan-cao, San-qi, Wu-wei-zi	0.33
Dang-gui, San-qi, Yan-hu-suo	0.42	Dang-gui, Gan-cao, Wu-wei-zi	0.33
Huang-qi, Mai-dong, Yan-hu-suo	0.42	Gan-cao, Wu-wei-zi, Yan-hu-suo	0.33
Gan-cao, Mai-dong, Wu-wei-zi	0.42	Chuan-xiong, Huang-qi, Wu-wei-zi	0.33
Dang-gui, Huang-qi, San-qi	0.41	Dan-shen, Huang-qi, San-qi	0.33
Chuan-xiong, San-qi, Yan-hu-suo	0.41	Chuan-xiong, Gan-cao, Ye-ge-gen	0.32
Huang-qi, Mai-dong, San-qi	0.41	Chuan-xiong, Ye-ge-gen, Yan-hu-suo	0.32
Mai-dong, San-qi, Yan-hu-suo	0.39	Dan-shen,Huang-qi, Wu-wei-zi	0.32
Dang-gui, Gan-cao, Mai-dong	0.39	Chuan-xiong, Dang-gui, Dan-shen	0.32
Dang-gui, Gan-cao, Yan-hu-suo	0.39	Dan-shen, Huang-qi, Yan-hu-suo	0.32
Dang-gui, Huang-qi, Wu-wei-zi	0.38	Dan-shen, Gan-cao, Yan-hu-suo	0.31
Chuan-xiong, Huang-qi, Mai-dong	0.38	Chuan-xiong,Dan-shen, Gan-cao	0.31
Huang-qi, San-qi, Wu-wei-zi	0.38	Chuan-xiong, Dan-shen, Huang-qi	0.31
Chuan-xiong, Dang-gui, Yan-hu-suo	0.38	Dan-shen, Gan-cao, San-qi	0.31
Chuan-xiong, Huang-qi, Yan-hu-suo	0.38	Chuan-xiong,Mai-dong, Yan-hu-suo	0.31
Mai-dong, Wu-wei-zi, Yan-hu-suo	0.38	Chuan-xiong, Huang-qi, Ye-ge-gen	0.31
Gan-cao, Mai-dong, Yan-hu-suo	0.38	Dan-shen, Gan-cao, Mai-dong	0.31
Huang-qi, Wu-wei-zi, Yan-hu-suo	0.38	Dang-gui, Mai-dong, San-qi	0.31
Dang-gui, Mai-dong, Wu-wei-zi	0.37	San-qi, Ye-ge-gen, Yan-hu-suo	0.31
Mai-dong, San-qi, Wu-wei-zi	0.37	Gan-cao, Huang-qi, Ye-ge-gen	0.3
Chuan-xiong, Huang-qi, San-qi	0.37	Bing-pian, Huang-qi, Yan-hu-suo	0.3
Dang-gui, Dan-shen, Huang-qi	0.37	Dang-gui, Dan-shen, Yan-hu-suo	0.3
Dang-gui, Gan-cao, San-qi	0.37	Bing-pian, Chuan-xiong, Yan-hu-suo	0.3
Gan-cao, Mai-dong, San-qi	0.37	Dang-gui, Dan-shen, Mai-dong	0.3
Dan-shen, Gan-cao, Huang-qi	0.37	Dan-shen, Mai-dong, Wu-wei-zi	0.3

**Table 6:** Frequent set *L*_4_.

Combination of TCM (4)	Confidence	Combination of TCM (4)	Confidence
Gan-cao, Huang-qi, Mai-dong, Wu-wei-zi	0.41	Dang-gui, Huang-qi, Mai-dong, Yan-hu-suo	0.32
Gan-cao, Huang-qi, San-qi, Yan-hu-suo	0.4	Chuan-xiong, Huang-qi, Mai-dong, Wu-wei-zi	0.32
Dang-gui, Gan-cao, Huang-qi, Mai-dong	0.38	Gan-cao, Mai-dong, San-qi, Wu-wei-zi	0.32
Huang-qi, Mai-dong, Wu-wei-zi, Yan-hu-suo	0.37	Gan-cao, Mai-dong, Wu-wei-zi, Yan-hu-suo	0.32
Gan-cao, Huang-qi, Mai-dong, Yan-hu-suo	0.37	Chuan-xiong, Gan-cao, Huang-qi, Mai-dong	0.32
Huang-qi, Mai-dong, San-qi, Yan-hu-suo	0.37	Chuan-xiong, Gan-cao, San-qi, Yan-hu-suo	0.32
Dang-gui, Huang-qi, San-qi, Yan-hu-suo	0.37	Dang-gui, Gan-cao, Huang-qi, Wu-wei-zi	0.32
Gan-cao, Huang-qi, Mai-dong, San-qi	0.36	Chuan-xiong, Dang-gui, San-qi, Yan-hu-suo	0.32
Dang-gui, Huang-qi, Mai-dong, Wu-wei-zi	0.36	Chuan-xiong, Gan-cao, Huang-qi, Yan-hu-suo	0.31
Huang-qi, Mai-dong, San-qi, Wu-wei-zi	0.36	Dang-gui, Huang-qi, Mai-dong, San-qi	0.31
Chuan-xiong, Dang-gui, Gan-cao, Huang-qi	0.35	Chuan-xiong, Dang-gui, Huang-qi, Mai-dong	0.31
Mai-dong, San-qi, Wu-wei-zi, Yan-hu-suo	0.34	Dang-gui, Dan-shen, Gan-cao, Huang-qi	0.31
Huang-qi, San-qi, Wu-wei-zi, Yan-hu-suo	0.34	Chuan-xiong, Dang-gui, Huang-qi, Yan-hu-suo	0.31
Dang-gui, Gan-cao, Huang-qi, Yan-hu-suo	0.34	Dang-gui, Gan-cao, Mai-dong, Wu-wei-zi	0.31
Gan-cao, Huang-qi, San-qi, Wu-wei-zi	0.33	Dan-shen, Huang-qi, Mai-dong, Wu-wei-zi	0.3
Gan-cao, Huang-qi, Wu-wei-zi, Yan-hu-suo	0.33	Chuan-xiong, Huang-qi, Mai-dong, Yan-hu-suo	0.3
Dang-gui, Gan-cao, San-qi, Yan-hu-suo	0.33	Chuan-xiong, Gan-cao, Huang-qi, San-qi	0.3
Chuan-xiong, Huang-qi, San-qi, Yan-hu-suo	0.33	Chuan-xiong, Dang-gui, Huang-qi, San-qi	0.3
Gan-cao, Mai-dong, San-qi, Yan-hu-suo	0.33	Dan-shen, Gan-cao, Huang-qi, Mai-dong	0.3
Dang-gui, Gan-cao, Huang-qi, San-qi	0.33	Gan-cao, San-qi, Wu-wei-zi, Yan-hu-suo	0.3

**Table 7:** Frequent set *L*_5_.

Combination of TCM (5)	Confidence
Huang-qi, Mai-dong, San-qi, Wu-wei-zi, Yan-hu-suo	0.33
Gan-cao, Huang-qi, Mai-dong, San-qi, Yan-hu-suo	0.32
Gan-cao, Huang-qi, Mai-dong, Wu-wei-zi, Yan-hu-suo	0.32
Gan-cao, Huang-qi, Mai-dong, San-qi, Wu-wei-zi	0.32
Dang-gui, Gan-cao, Huang-qi, Mai-dong, Wu-wei-zi	0.31
Dang-gui, Gan-cao, Huang-qi, San-qi, Yan-hu-suo	0.3
Gan-cao, Huang-qi, San-qi, Wu-wei-zi, Yan-hu-suo	0.3

**Table 8:** Confidence of herbal pairs.

Herbs	Confidence
Huang-qi, Dang-gui	0.85
Huang-qi, Gan-cao	0.82
Huang-qi, Mai-dong	0.75
Huang-qi, Yan-hu-suo	0.75
Huang-qi, San-qi	0.74
Huang-qi, Dan-shen	0.72
Dang-gui, Gan-cao	0.72
Yan-hu-suo, San-qi	0.69
Huang-qi, Chuan-xiong	0.67
Gan-cao, Mai-dong	0.67
Huang-qi, Wu-wei-zi	0.67
Dang-gui, Mai-dong	0.66
Dang-gui, Yan-hu-suo	0.65
Mai-dong, Wu-wei-zi	0.65
Dang-gui, San-qi	0.64
Gan-cao, Yan-hu-suo	0.64
Gan-cao, San-qi	0.63
Gan-cao, Dan-shen	0.62
Dang-gui, Dan-shen	0.62
Mai-dong, Yan-hu-suo	0.61
Dang-gui, Chuan-xiong	0.60

**Table 9:** Active compound ingredient list.

Mol ID	Molecule name	OB (%)	DL	Herb
MOL000211	Mairin	55.37707338	0.7761	Huang-qi
MOL000239	Jaranol	50.82881677	0.29148	Huang-qi
MOL000296	Hederagenin	36.91390583	0.75072	Huang-qi
MOL000033	(3S,8S,9S,10R,13R,14S,17R)-10,13-dimethyl-17-[(2R,5S)-5-propan-2-yloctan-2-yl]-2,3,4,7,8,9,11,12,14,15,16,17-dodecahydro-1H-cyclopenta[a]phenanthren-3-ol	36.22847056	0.78288	Huang-qi
MOL000354	Isorhamnetin	49.60437705	0.306	Huang-qi
MOL000371	3,9-Di-O-methylnissolin	53.74152673	0.47573	Huang-qi
MOL000374	5′-Hydroxyiso-muronulatol-2′,5′-di-O-glucoside	41.71766574	0.69251	Huang-qi
MOL000378	7-O-Methylisomucronulatol	74.68613752	0.29792	Huang-qi
MOL000379	9,10-Dimethoxypterocarpan-3-O-*β*-D-glucoside	36.73668801	0.9243	Huang-qi
MOL000380	(6aR,11aR)-9,10-Dimethoxy-6a,11a-dihydro-6H-benzofurano[3,2-c] chromen-3-ol	64.25545452	0.42486	Huang-qi
MOL000387	Bifendate	31.09782391	0.66553	Huang-qi
MOL000392	Formononetin	69.67388061	0.21202	Huang-qi
MOL000398	Isoflavanone	109.9866565	0.29572	Huang-qi
MOL000417	Calycosin	47.75182783	0.24278	Huang-qi
MOL000422	Kaempferol	41.88224954	0.24066	Huang-qi
MOL000433	FA	68.96043622	0.7057	Huang-qi
MOL000438	(3R)-3-(2-Hydroxy-3,4-dimethoxyphenyl) chroman-7-ol	67.66747949	0.26479	Huang-qi
MOL000439	Isomucronulatol-7,2′-di-O-glucosiole	49.28105539	0.62065	Huang-qi
MOL000442	1,7-Dihydroxy-3,9-dimethoxy pterocarpene	39.04541112	0.47943	Huang-qi
MOL000098	Quercetin	46.43334812	0.27525	Huang-qi
MOL000358	Beta-sitosterol	36.91390583	0.75123	Dang-gui
MOL000449	Stigmasterol	43.82985158	0.75665	Dang-gui

## Data Availability

All the data used to support the findings of this study are available from the corresponding author upon reasonable request.

## Supplementary Materials

Supplementary Materials Table S1: the Pin-yin names and their corresponding Latin names are shown.
