# Supplementary material for: Detection of Herbal Combinations and Pharmacological Mechanisms of Clinical Prescriptions for Coronary Heart Disease Using Data Mining and Network Pharmacology
Source: Evid Based Complement Alternat Med. 2021 Oct 23;2021:9234984. doi: 10.1155/2021/9234984 (PMC8557045; doi:10.1155/2021/9234984)
Supplement: Supplementary Materials — Table S1: the Pin-yin names and their corresponding Latin names are shown. [file 9234984.f1.docx]

**Supplementary Material:**

Table S1. The Pin-yin names and their corresponding Latin names

| Herb Pinyin | Latin Name | Herb Pinyin | Latin Name |
| --- | --- | --- | --- |
| Gan-cao | Radix et Rhizoma Glycyrrhizae | Gua-lou | Trichosanthis Fructus |
| Huang-qi | Astragali Radix | Qing-hao | Artemisiae Annuae Herba |
| Dang-gui | Angelicae Sinensis Radix | Qiang-huo | Notopterygii Rhizoma et Radix |
| Chuan-xiong | Chuanxiong Rhizoma | Sha-ren | Amomi Fructus |
| Yan-hu-suo | Corydalis Rhizoma | Dan-pi | Moutan Cortex |
| San-qi | Radix et Rhizoma Notoginseng | Chai-hu | Bupleuri Radix |
| Mai-dong | Ophiopogonis Radix | Huang-bo | Phellodendri Chinensis Cortex |
| Dan-shen | Salviae Miltiorrhizae Radix et Rhizoma | Rou-gui | Cinnamomi Cortex |
| Ye-ge-gen | Puerariae Lobatae Radix | Zi-shi-ying | Fluoritum |
| Wu-wei-zi | Schisandrae Chinensis Fructus | Hou-pu | Magnoliae Officinalis Cortex |
| Huang-lian | Coptidis Rhizoma | He-shou-wu | Polygoni Multiflori Radix |
| Bing-pian | Borneolum Syntheticum | Shu-di | Rehmanniae Radix Praeparata |
| Suan-zao-ren | Ziziphi Spinosae Semen | Ye-jiao-teng | Tuber Fleeceflower Stem |
| Huang-qin | Scutellariae Radix | Zhi-zi | Gardeniae Fructus |
| Gui-zhi | Cinnamomi Ramulus | Xing-ren | Armeniacae Semen Amarum |
| Gou-teng | Uncariae Ramulus Cum Uncis (Gouteng) | Zhi-qiao | Aurantii Fructus |
| Fu-ling | Poria | Ban-xia | Pinelliae Rhizoma |
| Ze-xie | Alismatis Rhizoma | Jue-ming-zi | Cassiae Seme |
| Shui-zhi | Hirudo | Da-huang | Rhei Radix et Rhizoma |
| Mu-xiang | Aucklandiae Radix | Xie-bai | Allii Bulbus Macrostemonis |
| Bai-shao | Paeoniae Radix Alba | Fu-zi | Aconiti Lateralis Radix Praeparaia |
| Ren-shen | Ginseng Radix et Rhizoma | Bai-zhu | Atractylodis Macrocephalae Rhizoma |
| Yin-yang-huo | Epimedii Folium | Dang-shen | Codonopsisradix |
| Sheng-di | Radix Rehmanniae | Xi-xin | Asari Radix et Rhizoma |
